# Accessing and Navigating Gender‐Affirming Hormone Therapy and Perspectives on Inclusive Pharmacy Care Among Transgender and Gender Diverse People: A Qualitative Study

**DOI:** 10.1111/hex.70951

**Published:** 2026-10-28

**Authors:** Jessica McCann, Sarah Wilcock, Ivy Dominy, Kayleigh Davison, Sarah Sharman, Anna Robinson‐Barella

**Affiliations:** ^1^ School of Pharmacy Newcastle University Newcastle upon Tyne Tyne and Wear UK; ^2^ NIHR Newcastle Patient Safety Research Collaboration (PSRC) Newcastle University Newcastle upon Tyne Tyne and Wear UK; ^3^ NIHR Greater Manchester Patient Safety Research Collaboration (PSRC) Manchester University Manchester Greater Manchester UK; ^4^ Patient and Public Contributor, School of Pharmacy Newcastle University Newcastle upon Tyne Tyne and Wear UK; ^5^ Horden Medical Practice Horden Peterlee County Durham UK; ^6^ School of Allied Health Professionals, Pharmacy, Nursing and Midwifery University of Sheffield Sheffield South Yorkshire UK

**Keywords:** community pharmacy services, gender affirming care, health inequities, pharmacy, qualitative, transgender persons

## Abstract

**Introduction:**

Transgender and gender diverse (TGD) people continue to experience inequities in access to gender‐affirming care, including stigmatisation, delayed treatment and non‐inclusive healthcare encounters. Community pharmacies may represent an accessible setting to support inclusive and affirming care; however, lived experiences of engaging with community pharmacy services in the context of gender‐affirming hormone therapy (GAHT) remain underexplored, particularly among younger people who represent the greatest proportion of the TGD community. This study aimed to explore the lived experiences of accessing, navigating and receiving GAHT, alongside participants' perspectives on barriers and facilitators to accessing inclusive and affirming care within community pharmacy.

**Methods:**

Semi‐structured interviews were conducted between October and November 2025. Interviews explored participant experiences of accessing GAHT and perspectives on inclusive and affirming pharmacy practice. Interviews were audio‐recorded, transcribed verbatim and analysed using reflexive thematic analysis. NVivo (Version 15) facilitated data management, and the COREQ and CCRF checklists informed reporting. Ethical approval was obtained from the Newcastle University Research Ethics Committee (reference: 66105/2023).

**Results:**

Ten participants, representing a range of gender identities, were interviewed. Three overarching themes were developed: (1) reflecting on the challenges of navigating gender‐affirming hormone therapy; (2) exploring the impact of healthcare barriers upon an individual and (3) accessing and receiving inclusive and affirming pharmacy care. Participants viewed community pharmacies as accessible and supportive environments for affirming care when underpinned by respectful communication, visible inclusivity, appropriate staff training and partnership working with TGD communities.

**Conclusion:**

In this study, TGD individuals described significant structural and interpersonal barriers when accessing GAHT in the United Kingdom, negatively influencing trust, wellbeing and engagement with healthcare services. Community pharmacies may represent an underutilised, yet important, setting for inclusive and gender‐affirming support. Embedding gender‐affirming training and co‐designed approaches within pharmacy services may strengthen accessibility, trust and equity in care for TGD communities.

**Lived Experience or Public Contribution:**

A person with lived experience from the TGD community (ID) acted as a co‐author and patient champion in this study; they supported the design and conduct of the research, advised on inclusive and non‐stigmatising approaches and supported preparation of the manuscript, contributing a lived‐experience perspective throughout.

## 1 Introduction

Transgender and gender diverse (TGD) individuals are a population that have historically faced, and continue to face, stigmatisation and marginalisation [1]. These individuals have experienced higher levels of health inequity compared to their cisgender counterparts [2, 3]; specifically, evidence has demonstrated disparities in access to healthcare services, experiences of stigma within healthcare systems [4] and poorer health‐related outcomes, such as avoidance of care, delayed treatment and increased risk of suicidality [5, 6].

The World Professional Association for Transgender Health (WPATH) has defined the terms transgender and gender diverse as ‘people whose gender identity differs from the gender socially attributed to the sex they were assigned at birth’ [7]. Recent global estimates suggest approximately 0.6% of adults and 2.7% of children and adolescents identify as transgender and gender diverse [6]. The Office for National Statistics have estimated that TGD individuals account for 0.54% of the population of the United Kingdom [8]; however, in reality, these figures may significantly underestimate the true population size, due to the voluntary nature of the census, the exclusion of individuals under 16, possible misinterpretation of the gender identity question, and reluctance or fear to disclose this information [9]. Irrespective of this, an estimated 260,000 TGD individuals in the United Kingdom require healthcare that affirms their needs and identities through inclusive communication, person‐centred care and supportive care environments [2, 8, 9]; with 16–17 year olds and 18–29 year olds making up the greatest proportion of the TGD community [10].

One of the most reported sources of psychological distress amongst the TGD community is gender dysphoria [11]. Gender dysphoria has been defined in the Diagnostic and Statistical Manual of Mental Disorders (DSM‐5) as ‘the marked incongruence between one's experiences or expressed gender and the gender assigned at birth, which is associated with clinically significant distress or impairment’ [12]. Gender dysphoria may manifest as persistent discomfort with one's body, gender roles and secondary sex characteristics. For some individuals, this can create a desire to pursue interventions, both surgical and pharmacological, to align their physical characteristics with their gender identity [11, 12].

Gender‐affirming hormone therapy (GAHT) is a core component of gender‐affirming care and involves the administration of exogenous hormones to suppress unwanted secondary sex characteristics and promote those aligned with an individual's gender identity [13, 14]. GAHT regimens and desired physical outcomes vary across TGD populations, meaning that care should be person‐centred and tailored to the treatment goals and priorities for each individual [7, 15]. Within the United Kingdom, TGD individuals may access this care through the National Health Service (NHS) or *via* a private General Practitioner (GP). A referral through the NHS typically involves a patient presenting to their primary care NHS GP, who then refers them to a tertiary care NHS Gender Dysphoria Clinic (GDC) for GAHT, where approved prescriptions are sent to community pharmacies for treatment, and associated medicines information, to be supplied [16]. The NHS pathway is often associated with significant waiting periods, with projections indicating that waits could extend to 15 years, due to the rising demand for NHS gender dysphoria clinics without increased service capacity [17]. Alternatively, patients may choose to instead self‐refer to private gender clinics for assessment and GAHT initiation, where specialist assessment is self‐funded and private clinicians carry out prescribing [16]. Private pathways can allow for more immediate access to treatment; however, this comes at an additional financial cost [18, 19].

The 2020 Cass Review [20], commissioned by NHS England, is a review of gender identity services for children and young people in England that evaluated the safety and clinical governance of paediatric gender care. Outcomes of the review that were subsequently implemented by NHS England included: the use of puberty blockers within formal research studies rather than routine care; GAHT being initiated from the age of 16 years (if certain conditions were met, such as long‐standing, persistent gender incongruence); and the incorporation of specialist multidisciplinary team (MDT) input within patient care plans, including mental health and neurodevelopment safeguarding [20].

Previous literature has highlighted perceptions of mistrust, stigma and health‐related disparagement [21] that have been experienced when healthcare professionals have limited awareness or competence in inclusive communication [9] or gender‐affirming care [18]. Instances of misgendering, deadnaming (being addressed by a previous name that no longer aligns with an individual's gender identity), fear of dismissal and inappropriate questioning have also resulted in the disempowerment of diverse gender identities [2]. These experiences have led to longstanding perceptions of mistrust amongst the TGD community and have reinforced inequities in accessing GAHT. The anticipation of negative healthcare interactions has been associated with TGD individuals self‐sourcing medication or avoiding treatment altogether [22], which can lead to poorer health outcomes through the administration of unregulated hormones, causing psychological distress and leading to a greater risk of suicidality [23, 24]. Consequently, the WPATH has advocated for more accessible frameworks for GAHT provision, such as the Informed Consent Model [25], to enable individuals to access GAHT through primary healthcare settings and removing the need for a psychiatric diagnosis [7].

Community pharmacy has been regarded as one of the most accessible primary healthcare settings in the United Kingdom, with pharmacy teams operating within an increasingly expanding scope of practice [15, 26]. Community pharmacy may provide an important point of contact through dispensing and supply of prescribed GAHT medicines, medicines‐related information and counselling, identification of medicines‐related problems, support with adherence and continuity of treatment and signposting or referral where appropriate [27]. The integration of gender‐affirming and inclusive care into pharmacy practice aligns closely with the *Standards for Pharmacy Professionals* set by the General Pharmaceutical Council (GPhC), which emphasises person‐centred care, effective communication and maintaining trust [28]. In response to The Cass Review, the Royal College of Pharmacy have published a position statement setting out key expectations and recommendations for pharmacists delivering GAHT‐related care [29]. These include: the facilitation of referrals to appropriate services, such as mental health support; the provision of appropriate education regarding medication options and uninterrupted access to necessary medications during transitions of care (e.g., between primary, secondary and tertiary care settings); and collaboration with health providers and policymakers to deliver safe and effective pharmaceutical care and reduce and prevent unregulated GAHT provision.

Alongside pharmacists, the wider pharmacy team play an important role in fostering positive patient experiences by creating inclusive pharmacy environments through respectful communication and awareness of GAHT‐related medicines [30]. Furthermore, pharmacy teams serve as a point of contact for broader non‐pharmacological interventions, such as supporting and signposting to additional services [31] and offering resources aimed at reducing cardiovascular health risk (like smoking cessation and lifestyle modification) for TGD individuals who use GAHT [32].

Evidence concerning the potential role of community pharmacy teams in supporting people accessing GAHT remains limited within the UK. Shedding light on the voices of those with lived experience of GAHT can showcase the barriers faced when accessing treatment. Further, lived experiences can then inform suggestions on the delivery of accessible and inclusive pharmacy care for TGD individuals.

## 2 Aim and Objectives

This study aimed to explore (i) the lived experiences of accessing, navigating and receiving gender‐affirming hormone therapy and (ii) participants’ perspectives on barriers and facilitators to accessing inclusive and affirming care within community pharmacy.

## 3 Methods

### 3.1 Recruitment and Sampling

The consolidated criteria for reporting qualitative research (COREQ) checklist [33] and the GRIPP2 framework of patient and public involvement in research [34] were used for the reporting of this study (see Supporting Information S1: Files 1 and 2). Research inclusion efforts were aligned with the Culturally Competent Research Framework [35, 36]. Considering the potential of digital strategies to facilitate qualitative research recruitment, a digital infographic was shared across professional social media accounts of the research team (S.W., J.M.C. and A.R.‐B.) and LGBTQIA+ university organisations across the United Kingdom. Interested individuals were asked to contact the researcher (S.W.) *via* email to receive a participant information sheet and consent form; those who provided their written consent were enroled in the study. There was no previous relationship established between the researcher and participants before the study commencement or recruitment. Participants did not receive financial compensation or reimbursement for taking part in this study.

Inclusion criteria comprised: participants aged 18 years or older, who were enroled in undergraduate or postgraduate study at a UK‐based university and who were currently accessing, or had previously accessed, GAHT in the United Kingdom. Trends over time have indicated that a considerable proportion of individuals accessing or in receipt of GAHT are adolescents or younger adults [10, 37]; with 16–17 year olds and 18–29 year olds making up the greatest proportion of the TGD community in the United Kingdom [10]. Reasons for this have previously been linked to higher levels of social awareness, acceptance and understanding of gender identity amongst these age ranges [38, 39]. Students and younger adults are also frequent users of community pharmacy services in the United Kingdom [40], making them a key population to investigate experiences and perspectives of GAHT access and explore how community pharmacies can provide inclusive and gender‐affirming care.

Purposive sampling aimed to ensure a diverse range of gender self‐descriptions, ages and ethnic backgrounds across the study population. Recruitment continued until sufficient data richness was achieved, informed through reflexive engagement with the depth and diversity of the dataset [41]. Ethical approval for this study was obtained from Newcastle University Research Ethics Committee (reference: 66105/2023).

### 3.2 Data Collection

Semi‐structured interviews were conducted by (S.W.), a cisgender female master's student researcher) between October and November 2025 and overseen by researchers (J.M.C. and A.R.‐B.) with experience in qualitative research. All participants were offered a choice of interview format (in‐person or remote), with all choosing interviews conducted remotely using Microsoft Teams. Our considerations for remote data collection echoed those of the wider literature [42, 43], namely, to facilitate participant safety and comfort and to allow diversity of inclusion opportunities across multiple geographical areas within the United Kingdom. A semi‐structured interview topic guide (see Supporting Information S1: File 3), based on previous work by the research team [44, 45, 46], was co‐developed with a member of the TGD community (I.D.) who acted as a patient champion and is co‐author in the study); this approach informed choices in language and phrasing of questions to ensure inclusivity and non‐stigmatisation. The semi‐structured interview topic guide centred around the key issues identified in the existing wider literature [9, 18, 23, 47], including: understanding participants’ journeys to accessing GAHT; experiences of engaging with GAHT through community pharmacies; and suggestions for promoting gender‐affirming pharmacy practice.

### 3.3 Data Analysis

All interviews were audio‐recorded and transcribed using Microsoft Teams; these were checked for accuracy by author (S.W.). Audio files were encrypted and were immediately deleted following transcription processes. All data were anonymised at the point of transcription, by assigning non‐identifiable pseudonyms, for example, participant 1, and so forth. Participants did not provide comment on the transcripts nor provide feedback on results.

The data were analysed using Braun and Clarke's reflexive thematic analysis [48, 49], and the principle of constant comparison guided an iterative process of data analysis (S.W., J.M.C. and A.R.‐B.). A detailed and thorough reading of the transcripts enabled data familiarisation. Initial descriptive codes were assigned to the data in a systematic process across the data sets, which were subsequently sorted into common coding patterns, enabling the development of primary themes from the data. The overarching themes and related subthemes were reviewed, refined and named once coherent and characteristic, and consensus was reached with the wider research team (J.M.C., I.D., S.S., K.D. and A.R.‐B.). The reflective process of data analysis was enhanced by post‐interview field notes, which enabled the researchers to work iteratively and consistently between interviewing participants and analysing data. NVivo (Version 15) software was used to facilitate data management. Data sufficiency and information power was determined by the research team after conducting nine semi‐structured interviews and thus, study recruitment ceased following interview number 10 [50].

### 3.4 Considerations When Reporting Participant Demographics

The researchers considered whether there were any associations between perspectives shared by participants and their demographics, including age, gender identity and ethnicity. Collecting sensitive personal information, such as gender and ethnicity, is complex as these protected characteristics are multifaceted and subjective [51]. TGD individuals are at a greater risk of experiencing marginalisation and stereotyping than their cisgender counterparts [9]. Efforts have been taken by the researchers to minimise these aforementioned risks when collecting and reporting participant demographics in this study; these include asking individual participants to express their own pronouns and gender identities through self‐reporting, in place of categorising identities.

## 4 Results

Ten participants were recruited and interviewed for this study (participant characteristics are described in Table 1). Participants self‐identified across a range of gender identities, including ‘trans man’ (*n* = 2), ‘trans woman’ (*n* = 2), ‘trans feminine’ (*n* = 2), ‘trans’ (*n* = 1), ‘non‐binary’ (*n* = 1), ‘trans queer man’ (*n* = 1) and ‘female’ (*n* = 1). The mean age of participants was 21 years (SD ± 1.6). The participants resided in several regions of the United Kingdom, including Greater London (*n* = 2), North East England (*n* = 3), West Midlands (*n* = 2), West Scotland (*n* = 2) and North West Wales (*n* = 1). Participants also reported multiple routes of access to GAHT: four participants obtained hormones exclusively through private clinics, two had self‐sourced their hormone therapy, and four used a combination of private and self‐sourced pathways. None of the participants were in receipt of hormones through NHS services at the time of interview. There were no refusals to partake, participant dropouts or repeat interviews and the average interview duration was 41.4 min (SD ± 5.8).

**Table 1:** Participant characteristics.

Participant number	Age range (years)	Self‐reported ethnicity	Region of residence within the UK	Self‐reported gender identity	Self‐reported preferred pronouns	Hormonal agents accessed	Route of access to GAHT
1	18–19	‘White, British’	Northeast England	‘Trans woman’	‘She/Her’	Oestrogen and anti‐androgen	Private, self‐sourced
2	20–21	‘White, British’	Greater London	‘Trans man’	‘He/Him’	Testosterone	Private
3	22–23	‘White, Scottish’	West Scotland	‘Trans feminine’	‘She/They’	Oestrogen and anti‐androgen	Private, self‐sourced
4	22–23	‘White’	West Scotland	‘Trans woman’	‘She/Her’	Oestrogen and anti‐androgen	Private, self‐sourced
5	18–19	‘White, British’	West Midlands	‘Trans man’	‘He/Him’	Testosterone	Private
6	22–23	‘White’	Northeast England	‘Trans feminine’	‘She/Her’	Oestrogen and anti‐androgen	Self‐sourced
7	18–19	‘White, British’	Northwest Wales	‘Non‐binary’	‘They/Them’	Testosterone	Private
8	22–23	‘White’	Greater London	‘Trans’	‘He/Him’	Testosterone	Private
9	20–21	‘White, British’	Northeast England	‘Female’	‘She/Her’	Oestrogen	Self‐sourced
10	22–23	‘White, British’	West Midlands	‘Trans queer man’	‘He/It’	Testosterone	Private, self‐sourced

Three overarching themes were developed that captured the participants’ lived experiences in accessing and engaging with gender‐affirming hormone therapy, as well as their reflections and recommendations for the provision of inclusive and gender‐affirming pharmacy care. These themes focused on [1] reflecting on the challenges of navigating gender‐affirming hormone therapy [2]; exploring the impact of healthcare barriers upon an individual, and [3] accessing and receiving inclusive and affirming pharmacy care (Figure 1). The three themes, and subthemes, are discussed in turn.

**Figure 1:**
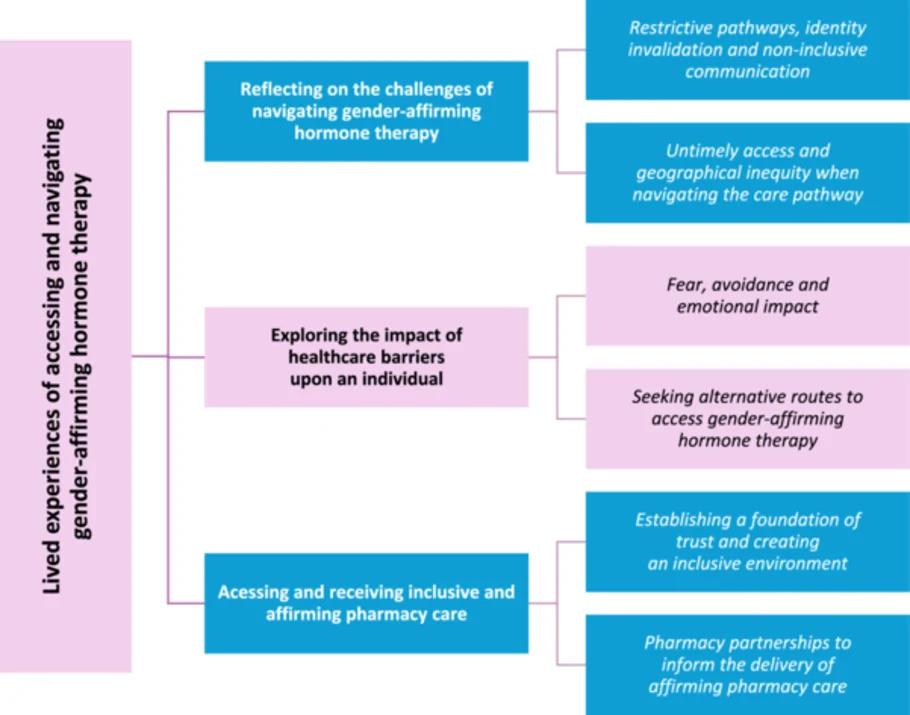
Lived experiences of accessing and navigating gender‐affirming hormone therapy: Developed from the perspectives of members of the transgender and gender diverse community.

### 4.1 Theme 1: Reflecting on the Challenges of Navigating Gender‐Affirming Hormone Therapy

Even where participants had accessed specialist gender‐related services, their experiences of care were not uniformly experienced as affirming or individualised.

#### 4.1.1 Restrictive Pathways, Identity Invalidation and Non‐Inclusive Communication

All participants shared their own lived experiences of accessing GAHT, with many negatively reflecting on certain procedures and consultation requirements currently embedded within NHS and private clinical pathways. For instance, participants described their experiences of undergoing psychiatric assessments as ‘mentally harmful and psychologically invasive’ (Participant 3) and perceived discussions around fertility preservation during pre‐treatment appointments as unwanted; however, to receive hormone therapy, participants felt they ‘had no choice’ other than to comply with these procedures in order to access and receive hormone therapy (Participant 1).

They [private GAHT provider] would not prescribe me anything until I had fertility storage… that wasn't something I was personally wanting, but I did it just so I could actually get my [hormone therapy] prescribed.

(Participant 3)

Participants also described negative past experiences relating to gender identity invalidation whilst accessing hormone therapy. Several reported that they felt pressure to present themselves in a defined and ‘traditional binary way… just to be taken seriously’ (Participant 4) when attending appointments (Participant 8). Participapants described feelings of expectations to conform to cisnormative ideals which left them unable to express their individual, authentic identities within healthcare settings.

I have other outfits set aside for things like healthcare interactions, because if I went in dressed how I do day to day, they don't take me seriously… they told me that there's no way I'm a trans man because I'm far too feminine.

(Participant 10)

Experiences of discrimination, stigma and non‐inclusive communication were also attributed as barriers in accessing GAHT. Participants reported experiences of misgendering (using a name, pronoun or title that does not align with their affirmed gender identity), deadnaming (the act of calling a transgender or non‐binary person by their birth name or a former name they no longer use, without their consent) and non‐gender‐affirming communication, which led to them feeling that their identities were misunderstood. They expressed that these experiences were distressing and ‘unfortunately really common’ (Participant 3). One participant shared ‘it's really awful … sometimes it feels like people are trying to undermine your gender on purpose and it feels really painful’ (Participant 7), while others reflected that these interpersonal barriers may reflect gaps in staff knowledge and training.

I got a call from a [GP] receptionist who just kept deadnaming me. I don't think it was deliberate prejudice or whatever, I think maybe she just wasn't trained on how to talk to trans people, which is a huge shame.

(Participant 2)

#### 4.1.2 Untimely Access and Geographical Inequity When Navigating the Care Pathway

Participants described the impact of long waiting times to access the NHS gender identity services and highlighted the ‘major barrier, geographically, is around [hormone treatment] access’ (Participant 8). One participant shared that ‘especially up in [name of Scottish city], I've heard the wait list is currently sat beyond the human lifetime’ (Participant 4). At the time of their interview, of the seven participants on waiting lists of up to 5 years, only one had received an initial assessment, and none had accessed hormone treatments through NHS pathways. The extended waiting periods fostered a sense of hopelessness, with one participant describing how they were ‘aware the NHS existed and did provide it [GAHT], but I also knew waiting lists for it are so horrendously long that it was basically not an option for me’ (Participant 4).

Geographical disparity in timely and effective GAHT care was flagged as another factor that influenced participants’ overall perception of the current GAHT care pathway. Some participants reported that, by living in certain areas within the UK (South of England), they were ‘in a very fortunate position as there was a GP practice near me that was listed on a map of places that would do informed consent prescriptions [for GAHT]’ (Participant 2). Another described how ‘going to the gender hormone clinic in [Greater London] meant I could get what [care] I needed pretty easily’ (Participant 8). Contrastingly, in other regions of the UK (Northwest Wales), a participant expressed experiences of limited access to GP surgeries that have a ‘good reputation with trans healthcare… there's only a couple of them but they're all full’ (Participant 7). The same participant also reflected on challenges with accessing shared care agreements when they ‘got in touch with my GP … I think I had to do some kind of form or something and I said, “Is there possibility of shared care?’ And they said ‘nope, we do not offer that”’ (Participant 7).

### 4.2 Theme 2: Exploring the Impact of Healthcare Barriers Upon an Individual

#### 4.2.1 Fear, Avoidance and Emotional Impact

Due to their previous negative experiences when accessing and engaging with GAHT, many participants described becoming fearful of engaging with healthcare professionals. Several termed the process of approaching healthcare professionals as one that was ‘terrifying’ (Participant 1) and with them ‘anticipating dismissal’ (Participant 10). Others described how barriers around fear and lack of trust within healthcare settings often led to health‐seeking avoidance; as a result, many participants shared concerns about timely and appropriate access to, and receipt of, GAHT.

Participants described feelings of frustration, hopelessness and exhaustion as they navigated GAHT access. Several expressed how ‘it's just so disheartening that you've gone looking for help and then just told “there's nothing we can do right now, we'll sit you on our waitlist for seven years”’ (Participant 1). The heightened anxiety associated with these interactions left some participants feeling that the process was detrimental to their mental health and, in some cases, intensified the effects of gender dysphoria.

They [healthcare professionals] are just leaving people on the side … you're kinda stuck in the middle trying to find help and you're slowly drowning.

(Participant 6)

#### 4.2.2 Seeking Alternative Routes to Access Gender‐Affirming Hormone Therapy

Participants highlighted a lack of guidance and support when navigating GAHT pathways, with some describing a sense of isolation and feeling like ‘you're on your own’ throughout the process (Participant 3). To mitigate this, participants discussed turning to online communities and forums to seek alternative guidance from others, as well as routes to access hormone treatments. One of these routes was through private GAHT providers, which was accessed by eight of the ten participants in this study. Described by one participant, private clinics offered them timely access to ‘life‐saving hormones’ (Participant 8) however, this came at a substantial financial cost. Some participants outlined the personal sacrifices that may be required to access private care, including taking on extra work, cutting essential expenses and relying on financial support to cover treatment expenses. One reflected on the socioeconomic inequity in alternative treatment options, ‘I was fortunate and privileged enough to be able to deal with that [financial] barrier, but not everyone will be’ (Participant 4).

It was a real matter of anxiety, like where am I going to get this money for hormones? I didn't know what my financial situation would be like at university … I was worried about how spending a lot of money on hormones would impact that.

(Participant 7)

Despite valuing the shorter waiting times to access hormone therapy privately, participants reflected on experiences of non‐inclusive, non‐affirming support and advice. Some described receiving medication with limited guidance, particularly regarding administration instructions. For instance, one participant explained that their private practitioner recommended they consult a primary care clinician for guidance around injection technique, but due to discomfort discussing TGD‐related care with that provider, they attempted to learn independently: ‘I'm not comfortable talking to GPs about being trans, so I just tried to learn on my own’ (Participant 2). Another participant described that the absence of specific injection guidance left them feeling unprepared and anxious, particularly when they experienced unexpected side effects: ‘the first time of using it [testosterone]… I got a bit of a shock because I didn't expect the injection site to hurt so much afterwards’ (Participant 5).

Many participants described choosing self‐sourced hormone therapy when both NHS and private pathways felt inaccessible or unattainable. The term self‐sourced, or ‘do it yourself’ or ‘DIY’, was commonly used to describe the use of self‐sourced hormone therapy without formal medical supervision. Participants described sourcing hormones from overseas pharmacies or purchasing raw ingredients from industrial suppliers to make their own medication. Despite understanding the risks, this pathway was perceived as essential and, for one participant, represented ‘the difference between a life of depression and suicide, and being a lot happier being myself’ (Participant 9). Participants highlighted the breadth of online community advice available surrounding DIY use and approaches to minimise potential harm from self‐sourcing, however, the emotional burden of using self‐sourced hormone therapy was also evident. Participants described the underlying fear and anxiety associated with undergoing treatment without clinical support.

You're on your own… I've really left like I've been on my own, which is terrifying … it would have felt good if there was support or resources available to help.

(Participant 7)

### 4.3 Theme 3: Accessing and Receiving Inclusive and Affirming Pharmacy Care

Participants’ experiences of navigating GAHT prompted discussion about what they would find affirming and inclusive in other healthcare encounters, including those from community pharmacy teams.

#### 4.3.1 Establishing a Foundation of Trust and Creating an Inclusive Environment

Due to previous experiences of fear, stigma and dismissal, participants emphasised the importance of establishing trust and respect within gender‐affirming care consultations. For participants, pharmacy staff that demonstrated ‘a level of empathy and extending the same level of respect that they would to a cis person’ was deemed essential in establishing a foundation of trust and approachability (Participant 10). A desire for community pharmacies to represent supportive and safe spaces was highlighted by several participants; many proposed the use of visible inclusion measures by pharmacy staff, such as wearing pronoun badges and displaying posters and trans health awareness campaign materials. One participant explained that ‘whenever an establishment has any sign of being supportive, it doesn't go unnoticed… it's nice knowing that you're somewhere where you can feel safe’ (Participant 1). Some participants also suggested that pharmacy services worked to incorporate preferred name and pronoun options into their digital systems to ensure that staff are equipped with the information needed to respectfully converse with their patients (without deadnaming or misgendering). However, many participants were wary that these measures could ‘be merely surface level’ and therefore must be reinforced and complemented with genuine gender‐affirming practice from pharmacy staff (Participant 9).

It's not as simple as expecting people to know your pronouns… but just asking or being able to put them into a system would really protect people from being accidentally hurt.

(Participant 6)

To promote consistent gender‐affirming practices in pharmacies, several participants highlighted a need to strengthen professional knowledge of TGD healthcare. Training could equip pharmacy teams with the confidence to provide inclusive and respectful gender‐affirming care for TGD individuals, including ‘how to speak to people about it [GAHT], how to access it… importantly how to use it safely’ (Participant 6). Participants envisioned that the combination of education, visible inclusion measures and systems that appreciated gender diversity could help shape pharmacies into approachable spaces for informed and inclusive support.

I think if pharmacy staff are trained more on trans healthcare, and the variety of genders of people they might encounter, it would help on an individual level… to know someone genuinely cares about you… but also in a broader way, ultimately, to create a healthcare system where you don't feel stigmatised.

(Participant 2)

#### 4.3.2 Pharmacy Partnerships to Inform the Delivery of Affirming Pharmacy Care

When discussing lived experiences and feelings of isolation, emphasis was placed on the importance of pharmacy team members being informed and non‐judgemental professionals, from whom TGD people could seek gender‐affirming care. Participants envisioned inclusive and welcoming pharmacy environments as accessible sources of support for people, regardless of whether they were navigating information seeking pathways or were in receipt of GAHT *via* NHS, private or DIY pathways. Specifically, participants suggested the role of pharmacy‐based support to include elements of signposting to appropriate gender‐affirming care resources and charity groups; providing education on hormone administration and regular monitoring; as well as offering a space where TGD people could seek inclusive health care without fear of stigma. Participants described pharmacies as being a combination of ‘access points to figure stuff out’ where they could ‘talk things out loud’ with the team members (Participant 8), alongside being a place of inclusive, affirming care. One participant described how ‘they [pharmacists] actually work with the medicines and know them inside out… and they're conceptually more approachable than a GP’ (Participant 1), inferring how subject expertise around medicines could offer unique opportunity for pharmacy teams to be more closely involved in the provision of gender‐affirming care.

To know that pharmacies were a place where you could get good medical information and not be chastised for using DIY… it would be reassuring to know that if there was a problem, there would be a health professional who would take you seriously and point out the best resources and stuff, because otherwise it's just friends or people on the internet.

(Participant 7)

Irrespective of their chosen GAHT pathway, all participants shared recommendations of mechanisms that could support the access, receipt and provision of inclusive and gender‐affirming care from pharmacy settings. An overarching theme in these recommendations centred on how collaborative partnership working, between pharmacy teams and those with lived experience of GAHT, would be essential in informing next steps. Participants were keen to ensure that any aspects of pharmacy service design or changes were informed by, and responsive to, the lived experiences of TGD individuals themselves.

Having joint resources, with pharmacies working with LGBTQ+ communities, could build safer spaces for people to have meaningful conversations without fear.

(Participant 6)

## 5 Discussion

This study contributes to the emerging evidence base concerning the experiences and perspectives of TGD people, and the role of community pharmacy teams in providing inclusive and affirming care. By exploring the perspectives of individuals who have navigated traditional and alternative routes of GAHT access in the United Kingdom, findings from this work have demonstrated how systemic barriers, non‐affirming encounters and perceptions of mistrust in healthcare services can influence access to, and receipt of, care. TGD people's experiences of navigating GAHT reveal structural, interpersonal and medicines‐related barriers that have implications for how community pharmacy services could be designed and delivered equitably; these findings illustrate potential for community pharmacy teams to deliver gender‐affirming care, along with suggested approaches and collaborations that seek to foster inclusive pharmacy practice.

Existing literature has highlighted the importance of authenticity for the mental wellbeing of TGD individuals, indicating that the ability to live and present authentically is associated with meaning, purpose and improved psychological health [52]. Authenticity has been recognised to support resilience against stigma and marginalisation [53]; our findings suggest that healthcare pathways that restrict authentic gender expression may undermine this protective measure and contribute to feelings of mistrust in healthcare services. Doubts about patient identity and legitimacy of GAHT prescriptions, has been previously linked with causing distress and delays in care for young people [54]. Inclusive and gender‐affirming pharmacy practice should, therefore, seek to accommodate diverse gender identities and support safe and authentic gender expression [55]; doing so may tackle barriers hindering engagement with treatment and build trusting provider‐patient relationships.

Experiences of stigmatisation within healthcare were deemed influential in shaping participant engagement with GAHT services. Accounts of misgendering, dismissive communication and pathologising assumptions undermined participants’ sense of safety in healthcare settings. Previous work, investigating the perspectives of transgender peoples’ interactions with pharmacies in Australia, reported that people often experience anxiety, avoidance behaviours and gender‐related challenges (*e.g*., misgendering) when accessing pharmacy services [56]. Consistent with existing literature [13, 18, 57], and alongside the model of minority stress [58, 59], this work demonstrates that non‐affirming encounters contribute to anticipatory anxiety, internalised stigma and avoidance of care among TGD individuals. Lewis et al. reported that transgender and gender‐nonconforming adults in the United States fear discrimination from pharmacies and often adopt avoidant or protective coping behaviours, such as delaying care or hiding their gender identity, because of previous negative experiences in pharmacy settings [60]. Our findings echoed the importance of healthcare practices that actively challenge stigmatisation [61].

Trusting relationships between patients and HCPs have been viewed as fundamental to safe and affirming care for TGD individuals [62]. In the absence of trust, people may be less likely to disclose concerns or seek support, increasing their vulnerability to unmet health needs [63]. This study offers insights into trust‐building approaches with community pharmacy teams. Wider pharmacy‐based research has further highlighted the importance of confidentiality and privacy in creating trustworthy spaces, such as offering discreet routes of access for individuals who feel unsafe in public‐facing environments [64, 65]. However, participants from this study and the wider literature cautioned that such approaches risk becoming tokenistic without genuine engagement with TGD communities and a clear understanding of their health needs [64]. Broader critiques of tokenism emphasise that meaningful inclusion requires coordinated and consistent affirming practice across the whole pharmacy team [66].

Pharmacy professionals have previously recognised a profession‐based willingness and commitment to providing trans healthcare [67], citing the importance of fostering accessible and culturally sensitive care in an inclusive environment for TGD individuals [68, 69]. However, previous works have also identified profession‐centred gaps in confidence and training related to TGD healthcare, suggesting that willingness alone may be insufficient to ensure the consistent delivery of affirming care [68]. Chaudhary et al. found that pharmacy staff felt rewarded when they established rapport and created safe spaces for their patients; however, they highlighted the need for communication training and education to improve their confidence in providing care for transgender people [70]. To translate this into safe and sustainable practice, our research suggests that gender‐affirming healthcare training be embedded within the undergraduate and post‐registration initial education and training of pharmacists, as well as training programmes for wider members of the pharmacy team. Previously, pharmacy education research has called for curricular diversification and training opportunities to build the cultural competence and cultural humility of healthcare professionals [44, 46, 68, 71]. Such training may include guidance on hormone monitoring, safe administration counselling and inclusive language frameworks, leveraging pharmacists’ and pharmacy teams’ scope of practice to improve gender‐affirming care delivery [15].

Collaborative co‐design principles were suggested as a method for designing and informing mechanisms for inclusive pharmacy‐based GAHT support, where views from those with lived experience could shape and inform meaningful change to service delivery. This echoes wider LGBTQIA+ healthcare literature in having the potential to positively influence levels of engagement with services and providers [72]. Embedding co‐design principles within affirming pharmacy care can reduce the risk of tokenistic inclusion by ensuring services remain responsive to TGD community needs [73]. Beyond this, researchers and pharmacy policy makers should seek to proactively collaborate with LGBTQIA+ organisations and third‐sector organisations to embed GAHT safety‐focused and affirming services within community pharmacy settings [74, 75]; such partnerships may also strengthen sexual health support through improved access to screening, safer sex advice and HIV prevention programmes [74, 75]. This collaborative and holistic approach to care could facilitate engagement and trust in both clinical and community‐based support systems.

Ten participants were purposively sampled and interviewed in this study to include a range of gender identities and experiences with the GAHT care pathway; however, we acknowledge that there are some limitations to this work. Perspectives and experiences shared in this work may not be generalisable to those within the entire TGD community. Due to the specific focus of this research question, data were obtained from university students aged 18 years and over who had either previously engaged with GAHT or who are currently undergoing GAHT. Future studies may seek to investigate the lived experiences and perspectives across different age groups of TGD individuals. Furthermore, all participants in this study self‐reported their ethnicity as White (British or Scottish); future qualitative investigations should seek to capture the views of TGD individuals from underrepresented and ethnically minoritised groups, being cognisant of the intersecting inequities between ethnicity, culture and transgender healthcare [76]. In addition, researchers may seek to explore the perspectives of the pharmacy team, alongside people with lived experiences, to gain further insight into the design, delivery and implementation of inclusive, pharmacy‐based gender‐affirming care.

### 5.1 Researcher Reflexivity and Positionality Statement

When conducting research into inequities and minoritised groups, it is important to acknowledge the reflexivity and positionality of the research team. Authors J.M.C. (a pharmacist and doctoral researcher in medication safety), S.W. (an undergraduate student pharmacist), K.D. (a pharmacist and early careers researcher in health literacy and health equity), S.S. (an academic Pharmacy Technician leading on equality, diversity and inclusivity in pharmacy education and practice, and researcher with particular focus on LGBTQIA+ populations) and A.R.‐B. (a pharmacist and qualitative researcher with a focus on tackling inequities within pharmacy services, access and receipt of healthcare and working with people from minoritised communities, and leader of equality, diversity and inclusivity in pharmacy education and practice) acknowledged their positionality in this field of research as cisgender individuals and role as allies in striving to promote health equity within pharmacy practice. Author I.D. self‐reported as transgender; they advocated for inclusivity, appropriateness and sensitivity throughout the research process and their lived experience contributed an important perspective that challenged, refined or contextualised our interpretations.

Importantly, the possibility of courtesy bias was considered, particularly given the professional background and expertise of the research team, as well as the potentially sensitive nature of discussing healthcare experiences. The interviewer emphasised that participation was confidential and that there were no ‘right or wrong’ answers to questions. Participant accounts were understood as situated subjective interpretations of their lived experiences, rather than objective representations.

## 6 Conclusion

This study contributes to the emerging evidence base concerning the experiences and perspectives of TGD people, and the role of community pharmacy in providing inclusive and gender‐affirming care. By exploring the perspectives of individuals who have navigated traditional and alternative routes of GAHT access in the United Kingdom, findings from this work have demonstrated how systemic barriers, non‐affirming encounters and mistrust in healthcare services can influence access to, and receipt of, care. These findings leverage pharmacists’ and pharmacy teams’ scope of practice to deliver inclusive and affirming pharmacy care, along with suggested approaches and collaborations that seek to foster inclusive pharmacy practice. Future studies should explore perspectives on GAHT access and how community pharmacies can improve inclusivity for TGD populations, across diverse age groups and ethnic backgrounds, to ensure equitable and affirming care. Future studies should seek to proactively collaborate with LGBTQIA+ organisations and third‐sector organisations to embed gender‐affirming services within community pharmacy settings, as well as examine the intersectionality between differing age groups and ethnicities, amongst the TGD community. Furthermore, research should examine diversification of the initial education and training approaches for pharmacy professionals to strengthen competence and confidence in providing care for TGD people.

## Author Contributions

**Jessica McCann:** conceptualisation, methodology, validation, writing – review and editing, supervision,; project administration, data curation, software, formal analysis, visualisation, investigation, resources. **Sarah Wilcock:** conceptualisation, investigation, writing – original draft, writing – review and editing, data curation, software, formal analysis, methodology, validation, visualisation. **Ivy Dominy:** validation, visualisation, writing – review and editing. **Kayleigh Davison:** writing – review and editing. **Sarah Sharman:** writing – review and editing. **Anna Robinson‐Barella:** funding acquisition, conceptualisation, investigation, methodology, validation, supervision, data curation, software, formal analysis, visualisation, resources, project administration, writing – review and editing.

## Funding

This work was undertaken as part of a Master of Pharmacy undergraduate degree (author S.W., supervised by authors J.M.C. and A.R.‐B.); there was no funding for this work.

## Ethics Statement

Ethical approval was obtained from the Newcastle University Research Ethics Committee (reference: 66105/2023). Participants were not recruited *via* the NHS.

## Conflicts of Interest

The authors declare no conflicts of interest.

## Supporting information


Supporting File

## Data Availability

The data that support the findings of this study are available from the corresponding author upon reasonable request.
